# Rapid saccadic categorization of other-race faces

**DOI:** 10.1167/jov.21.12.1

**Published:** 2021-11-01

**Authors:** Peter de Lissa, Nayla Sokhn, Sasha Lasrado, Kanji Tanaka, Katsumi Watanabe, Roberto Caldara

**Affiliations:** 1 Eye and Brain Mapping Laboratory (iBMLab), Department of Psychology, University of Fribourg, Fribourg, Switzerland; 2 Faculty of Arts and Science, Kyushu University, Fukuoka, Japan; 3 Faculty of Science and Engineering, Waseda University, Tokyo, Japan; 4 Faculty of Arts, Design, and Architecture, University of New South Wales, Sydney, Australia

**Keywords:** face processing, race, other-race face categorization advantage, eye movements, saccades

## Abstract

The human visual system is very fast and efficient at extracting socially relevant information from faces. Visual studies employing foveated faces have consistently reported faster categorization by race response times for other-race compared with same-race faces. However, in everyday life we typically encounter faces outside the foveated visual field. In study 1, we explored whether and how race is categorized extrafoveally in same- and other-race faces normalized for low-level properties by tracking eye movements of Western Caucasian and East Asian observers in a saccadic response task. The results show that not only are people sensitive to race in faces presented outside of central vision, but the speed advantage in categorizing other-race faces occurs astonishingly quickly in as little as 200 ms. Critically, this visual categorization process was approximately 300 ms faster than the typical button press responses on centrally presented foveated faces. Study 2 investigated the genesis of the extrafoveal saccadic response speed advantage by comparing the influences of the response modality (button presses and saccadic responses), as well as the potential contribution of the impoverished low-spatial frequency spectrum characterizing extrafoveal visual information processing. Button press race categorization was not significantly faster with reconstructed retinal-filtered low spatial frequency faces, regardless of the visual field presentation. The speed of race categorization was significantly boosted only by extrafoveal saccades and not centrally foveated faces. Race is a potent, rapid, and effective visual signal transmitted by faces used for the categorization of ingroup/outgroup members. This fast universal visual categorization can occur outside central vision, igniting a cascade of social processes.

## Introduction

Human diversity in physical appearance has been a source of contemplation and curiosity since the beginning of human history. Race is a universal, socially constructed concept used to categorize humans originating from different geographical locations by salient physiognomic variations (i.e., skin tone, eye shape). Interestingly, this visual categorization impacts upon face processing performance and social judgements. People are typically better at recognizing faces of their own race than those of other races, an effect coined as the other-race effect (Brigham & Barkowitz, 1978; Fallshore & Schooler, 1995; Meissner & Brigham, 2001; Rhodes et al., 2009; Valentine & Endo, 1992). Coupled with this identity recognition advantage is a seemingly counterintuitive speed advantage. When judging the race of a face people are quicker to categorize other- than same-race faces (Caldara et al., 2004; Feng et al., 2011; Ge et al., 2009; Levin, 1996, 2000; Liu-Shuang, Norcia, & Rossion, 2014; Valentine & Endo, 1992; Zhao & Bentin, 2008, 2011). Termed the other-race categorization effect (ORCA), there is evidence supporting a conceptual link between these face–race effects, where the strength of one reliably predicts the strength of the other (Ge et al., 2009). The prevailing theory accounting for the nature of these identity and race categorization effects is the face–space model proposed by Valentine, whereby faces are encoded through experience and stored in a multidimensional space (Valentine & Endo, 1992; Valentine, 1991). In particular, the speed advantage for other-race categorization is theorized to be due to a less elaborated face space for other-race faces. Other-race faces are less familiar and represent few exemplars encoded with suboptimal diagnostic information (i.e., the color of the eyes is not diagnostic in East Asian [EA] faces, but it is for Caucasian faces). As a result, other-race faces share distinctive features (i.e., same hair color), a lack of variance across exemplars, and will be grouped together in a distinct part of the face space. The high density across exemplars will engender a dense cluster that will lead to quicker judgements about the race of other-race faces, but convergingly a face identification impairment as the distance between exemplars is smaller. This face space explanation for the other-race effect and the ORCA has also been validated by computational models outlining the statistical nature of perceptual learning for faces (Balas, 2013; Caldara & Abdi, 2006; Furl, Phillips, & O'Toole, 2002; O'Toole et al., 1994). Race itself has also been proposed to represent a visual feature that is recognized in the early structural processing of faces (Levin, 1996, 2000). Visual expertise with same- and other-race faces would be shaped by the familiarity with such race features.

Critically, other-race effects are found across cultures, where the definition of an “other” race is solely dictated by which race a person is most familiar with (Sangrioli et al., 2005). Although a number a studies have found that there are cultural differences in where participants tend to fixate on faces when judging the identity of different races (Blais et al., 2008; Caldara, Zhou, & Miellet, 2010; for a review see Caldara, 2017), we do not yet have a clear understanding on how faces are processed outside of the central foveal visual area, because nearly the entire scientific literature has been established with stimuli presented in the fovea. This lack of knowledge is quite puzzling, because routinely natural vision involves the perception of faces outside of the fovea; very rarely do we only see faces directly from the central visual field. A rapid complex dynamic is necessary to integrate peripheral and foveal information in the visual system. The fast and initial low spatial frequency processing of faces in the peripheral visual field allows us to detect the diagnostic information necessary to generate predictions of the spatial arrangement of the faces and direct a saccade for a refined foveal analysis. The understating of this fine-grained interplay is necessary to have a comprehensive knowledge of face processing and human vision, but has been largely ignored. The sensitivity to faces presented in the periphery has recently been highlighted in saccadic choice paradigms where we are typically significantly faster to make saccades toward faces than to other objects (Boucart et al., 2016; Crouzet et al., 2010; Crouzet & Thorpe, 2011; Guyader et al., 2017; Kauffmann et al., 2019). Of particular note are the findings of Crouzet et al. (2010) showing that human face detection using a saccadic choice paradigm can occur within 100 ms after stimulus onset, suggesting a very early stage of perceptual processing to be responsible for such explicit judgements. Not only are people typically faster to make eye movements toward faces as a category, but we are also able to process an array of dimensions within faces when presented extrafoveally, such as gender (Ramon, Sokhn, & Caldara, 2019; Ramon, Sokhn, Lao, & Caldara, 2018), emotion (Bayle et al., 2011; Smith & Rossi, 2018) direction of eye gaze (Mares et al., 2016), and identity to a degree (Ramon et al., 2019; Visconti di Oleggio Castello & Gobbini, 2015). Self-guided saccadic eye movements may, therefore, be due in part to the perception of these dimensions in daily life and related to what we may choose as categorical targets for our attention and eye gaze. However, to the best of our knowledge, whether race categorization can be achieved in extrafoveal analysis of faces remains unknown. We, therefore, do not yet know whether race can modulate oculomotor behaviors in extrafoveal visual contexts and play a role in the selection of face targets to saccade toward. Some studies have obliquely moved toward shedding light onto the processing of race in visual contexts involving multiple potential target faces, with faster response times observed in a visual face search task when targets belonged to another race (Zhou et al., 2015), and differences relating to observer culture group when searching for faces of specific races (Sun et al., 2013). In contrast, an earlier study found a speed advantage for same-race faces in a similar task (Lipp et al., 2009), suggesting a somewhat inconsistent pattern in such tasks. However, these studies did not control for eye movements or specifically measure the processing of race in extrafoveal faces, because the responses in each task concluded a search period where each of the faces could be foveated upon and analyzed. The processing of race in extrafoveal vision is therefore still not clear, because a separation of prefixation and postfixation processing cannot be made with such experimental paradigms. Determining whether race can be categorized extrafoveally would therefore clarify whether such processing may occur before fixation. In addition, an analysis of minimum saccadic response time of such race-related judgments can offer valuable insight into whether earlier or later stages of perceptual processing are implicated in the categorization of race in extrafoveal vision, akin to the minimum saccadic response analyses performed by Crouzet et al. (2010).

To address these questions, we performed two categorization by race studies while controlling for the eye movements of Swiss Western Caucasian (WC) and Japanese (EA) observers. Using a cross-cultural sample further allowed us to investigate any cultural differences of which parts of the faces are fixated upon in the foveal and extrafoveal tasks. There is evidence suggesting that EA participants tend to make central fixations to faces during race categorization tasks, whereas WC participants tend to fixate the eye or mouth regions (Blais et al., 2008). Similar patterns have been found for face recognition tasks (Blais et al., 2008; Caldara, Zhou, & Miellet, 2010; Kelly et al., 2011; Miellet et al., 2010; Rodger et al., 2010), although other studies have found differences in fixation patterns based on the race of the face being viewed rather than the race of the observer (Goldinger et al., 2009; Or et al., 2015). In study 1, we used a saccadic choice task to establish whether and how race is processed extrafoveally and which facial features are fixated to achieve this feat. The results of study 1 showed that not only does race categorization reliably occur in extrafoveal vision, but that the ORCA effects in an extrafoveal saccadic response paradigm occur much earlier (approximately 300 ms) than in the typical central button press paradigm (approximately 600 ms). Study 1 also revealed that the well-established cultural fixation biases emerged only in the foveated condition and were abolished by extrafoveal saccadic responses. However, there are differences between the foveal key press and extrafoveal saccadic choice paradigms that do not make a direct comparison of the reactions times easy to interpret. Therefore, to more readily interpret the fast response times observed in the saccadic choice task in study 1, we implemented a second study to compare the effects of spatial frequency loss inherent in extrafoveal perception, as well as the use of button presses as a response modality in an extrafoveal presentation paradigm. In study 2, we replicated the foveal key press experiment, while applying a retinal filter to reconstruct the loss of visual acuity inherent in extrafoveal vision to determine whether the impoverished visual information was at the root of the saccadic response speed advantage observed in study 1. Second, to determine whether the fast response times observed in the extrafoveal saccadic choice paradigm were due to a difference in the response modality, we recreated the extrafoveal saccadic choice paradigm but required participants to make key press responses instead of saccades. In addition to this comparison, we further compared the extrafoveal key press response times with the foveal key press response times from study 1 to determine the effect of extrafoveal presentation when the response modality was the same. Study 2 revealed that the low spatial frequency typical of the extrafoveal visual information processing does not play a critical role in the fast saccadic response toward other-race faces, but the response modality does.

## Methods for study 1

The Human Ethics Committee at the University of Fribourg and the University of Waseda approved the methods and procedure used in this study. All participants provided written informed consent in accordance with the Declaration of Helsinki.

The data used in the statistical analyses and in the construction of the graphical depictions in studies 1 and 2 are openly available at doi: 10.17605/OSF.IO/PMKR3 (osf.io/pmkr3).

### Participants

Eighty participants in total took part in study 1 in two separate cross-cultural groups; 40 WC participants (34 female, mean age = 21.4 ± 2.2 years) of the University of Fribourg and 40 EA participants (25 female, mean age = 21 ± 2 years) of Waseda University and the surrounding region of Kanto, Japan. After data processing and exclusion procedures (see data analysis, preprocessing), 28 WC datasets and 31 EA datasets were included for statistical analysis in the extrafoveal saccadic choice task. All 40 of each WC and EA datasets were included in the foveal key press task. All participants had normal or corrected-to-normal vision, and gave their written informed consent before participating in the study. Participants were given either a monetary reward or course participation credits for their participation.

### Stimuli and procedure

Stimuli in study 1 consisted of grayscale images of 10 Caucasian and 10 Asian identities (equal numbers of male and female). The images were neutral expression frontal portrait photographs of Belgian (WC) and Chinese (EA) students aged between 18 and 25 years (database identities: AM1-5, AF1-5, CM1-5, and CF1-5), and have been used in previous studies investigating other-race behavioral effects (Michel, Caldara, & Rossion, 2006; Michel, Rossion, Han, Chung, & Caldara, 2006). The faces were cropped to exclude hair and ears, and were matched for amplitude spectra, luminance, and contrast using the SHINE toolbox (Willenbockel et al., 2010). The face stimuli were presented at a distance of 70 cm from the participants (subtending 9° × 12° of visual angle each) on VIEWPixx/3D (Switzerland) and EIZO FORIS FG2421 (Japan) LCD monitors (120 Hz refresh, 1920 × 1080 resolution). The luminance of the ViewPixx monitor was set at 100cd/m^2^ at full 255 intensity and the mean pixel intensity of the presented images was 114 (8-bit grayscale). Stimuli were presented via MATLAB software using PsychToolBox (Brainard, 1997; Kleiner et al., 2007; Pelli, 1997) and using an Eyelink extension to control the eye tracker (Cornelissen et al., 2002).

Study 1 involved two experimental paradigms where participants had to judge the race of presented faces. In the first paradigm, a single Caucasian or Asian face was presented in the center of the screen (spatially jittered between ±2° left and right) and participants (both WC and EA observers) were instructed to press an appropriate key button to categorize the faces by race (“k” for Caucasian and “a” for Asian, and then counterbalanced). Button press accuracy and reaction times were recorded for statistical analysis. The trials began with a central fixation cross for between 500 and 700 ms, followed by a brief black grey screen for 200 ms that was replaced by the face image. The face remained on the screen until a button press was recorded, which ended the trial (no time-out threshold). The centrally presented “foveal” presentation experiment consisted of 100 trials each of Caucasian and Asian faces for a total of 200 randomized trials.

**Figure 1.**
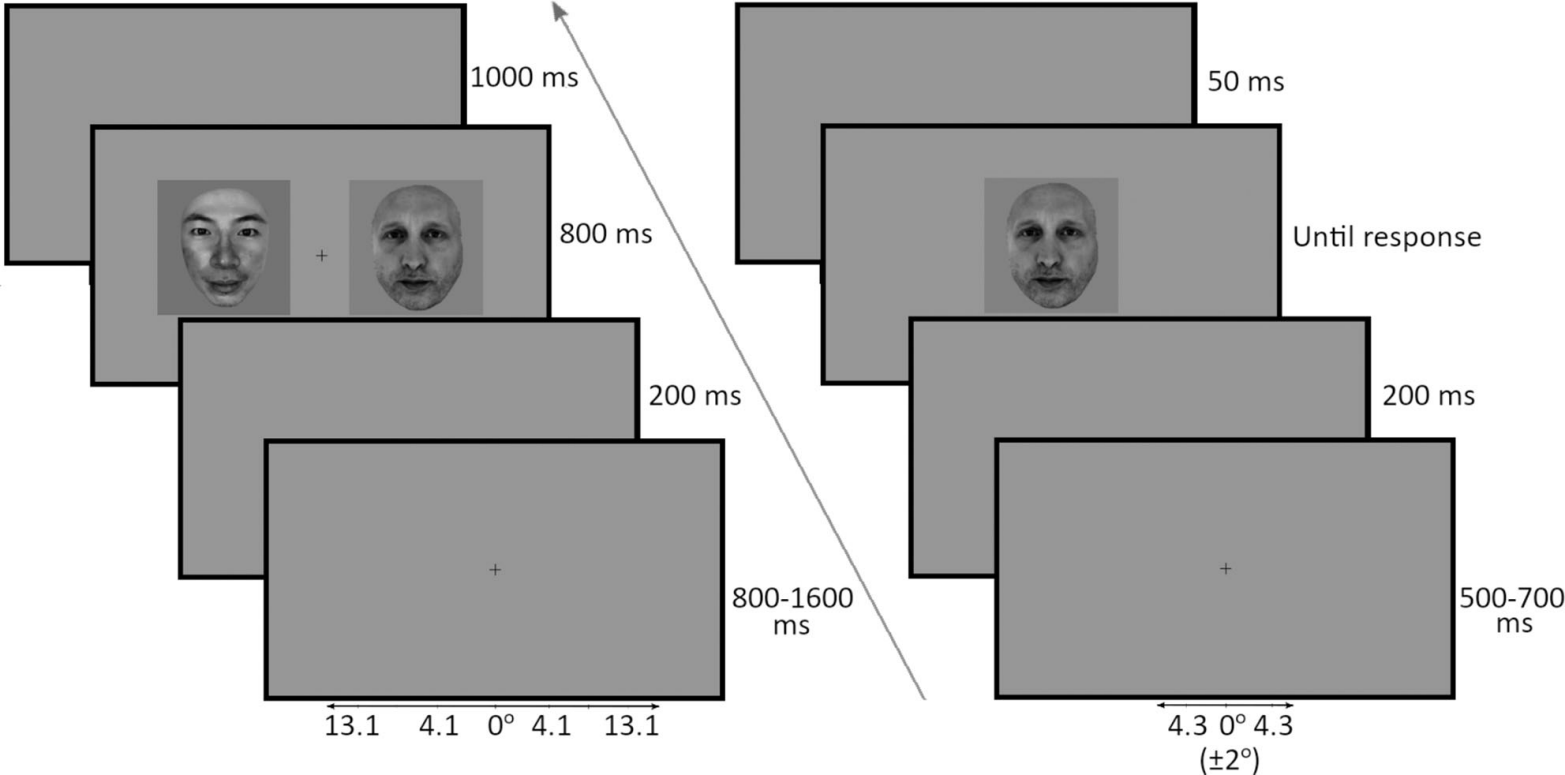
Examples of the appearance of the stimuli and trial sequences presented to participants in the extrafoveally presented saccadic choice (left) and foveally presented key press (right) tasks. The face identities depicted in this figure and those following were created by the authors, were not used in the study, and are for illustration purposes only. Permission to use these identities has been granted to the authors.

In the second experimental paradigm, a Caucasian and an Asian face were simultaneously presented in the left and right sides of the screen (left/right positioning was randomized), and participants were instructed beforehand to make an eye movement toward either the Caucasian or the Asian face. Owing to the use of this forced-choice task, the participants were instructed ahead of time whether to locate the Asian or the Caucasian faces through instructions given at the beginning of each new experimental block of 100 trials. The order of these blocks were counterbalanced between participants. The saccadic reaction accuracy (saccades leading into target faces) and reaction time (onset time of response saccades) were recorded for statistical analysis. The trials began with a central fixation cross displayed for between 800 and 1600 ms, followed by a brief grey screen for 200 ms that was replaced by Caucasian and Asian faces presented in the left and right sides of the screen, offset from the center by 8.6° (Figure 1). The faces remained on the screen for 800 ms, before being replaced by a blank grey screen for 1,000 ms. The saccadic choice task paradigm consisted of 100 trials each where the target was Caucasian and Asian, respectively, for a total of 200 randomized trials. Participants in study 1 performed both the foveal key press and the extrafoveal saccadic response experiments.

Eye position data were recorded during both tasks to allow for analyses of fixation location and duration on the stimuli during the respective tasks.

Eye positions were recorded through a desktop-mounted Eyelink 1000 (SR Research) monocular (left) eye tracker sampling at 1,000 Hz. Calibrations of the eye tracker (9 points) were performed at the beginning of the experiment, between blocks, and after breaks in the trials. Chin and forehead rests were used to stabilize participants' heads during the testing sessions. Saccadic response latencies were calculated for each trial, and fixation maps were constructed for each participant's fixation patterns on the face stimuli in each of the two tasks to investigate whether there were cross-cultural differences in sampling strategies.

### Data analysis

#### Preprocessing

We applied the algorithm developed by Nyström and Holmqvist (2010) to retrieve fixations and the onset of the first saccade in the extrafoveal saccadic choice task. Fixations were realigned in a normalized space using the iTemplate toolbox (Xiao & Lee, 2018). A total of 247 trials (2.09%) in which the onset of the first saccade was faster than 80 ms were removed from the analysis because they were considered as very early saccades (Visconti di Oleggio Castello & Gobbini, 2015). For the foveal key press task, the reaction time was recorded in each trial when a given behavioral response was provided. A total of 389 trials (2.43%) in which reaction time was higher than 2.5 standard deviations from the mean (within each subject) was discarded from the analysis as they were considered as outliers. Only correct trials of participants that were above chance level in both experiments were analyzed. A total of 28 WC and 31 EA participants were included in the analyses of the extrafoveal saccadic choice task and a total of 40 EA and 40 WC participants for the analyses of the foveal key press task.

#### Behavioral analysis

Reaction times of the WC and EA observers were analyzed with a linear mixed model using a gamma distribution. Two predictors were coded as dummy variables: the race of the stimuli (Caucasian stimuli used as reference stimuli) and the race of the observers (Caucasian group as reference group) with their interaction factor. Subject was included as a random intercept to account for repeated measures. The 2 × 2 design was conducted separately for the foveal-presentation (using key press reaction times as input data) and extrafoveal presentation (using saccadic response onset times as data input) paradigms.

(1) $$\begin{array}{c} Reaction\;time∼Race\times Group+\left(1|observers\right) \end{array}$$

Models were fitted in R (version 3.2.4; R Core Team, 2013) using the lme4 package (Bates et al., 2015) and the lmerTest (Kuznetsova et al., 2015) for null hypothesis testing. Figures were produced with the ggplot2 package (Wickham, 2016).

#### Fixation map analysis

The fixation duration maps were computed using the statistically data-driven method built in iMap4 (Lao et al., 2017). We then smoothed these maps with a two-dimensional Gaussian kernel function at 0.8° of visual angle by selecting the estimated option. This method computes for each condition and observer the expected values across trials. Finally, we normalized the maps by dividing them with the sum duration of each trial. A pixel-wise linear mixed model was then applied on the smoothed normalized fixation maps and a multiple comparison correction was conducted by using a bootstrap spatial clustering method (cluster size option) to control for type 1 errors. The design model chosen for the fixation map analysis involved fixation duration considered as the response variable, and group of observers considered as the predictor.

## Methods for study 2

The Human Ethics Committee at the University of Fribourg and the University of Waseda approved the methods and procedure used in this study.

### Participants

Data were collected from separate participant groups in two different foveal and extrafoveal experimental paradigms, each with cross-cultural samples. Forty participants were tested in a spatially filtered foveal paradigm: 20 WC participants (16 female, mean age = 22.4 ± 4.2 years) from the University of Fribourg and 20 EA participants (10 female, mean age = 21.1 ± 2.3 years) from Waseda University and the surrounding region of Kanto, Japan.

Forty participants were also tested in an extrafoveally presented key press paradigm: 20 WC participants (14 female, mean age = 21.4 ± 4.4 years) from the University of Fribourg and 20 EA participants (10 female, mean age = 20.9 ± 1.5 years) Waseda University and the surrounding region of Kanto, Japan.

All participants had normal or corrected-to-normal vision, and gave their informed consent before participating in the study. Participants were offered 50 CHF for their time or course participation credits.

### Stimuli, procedure, and data analysis

**Figure 2.**
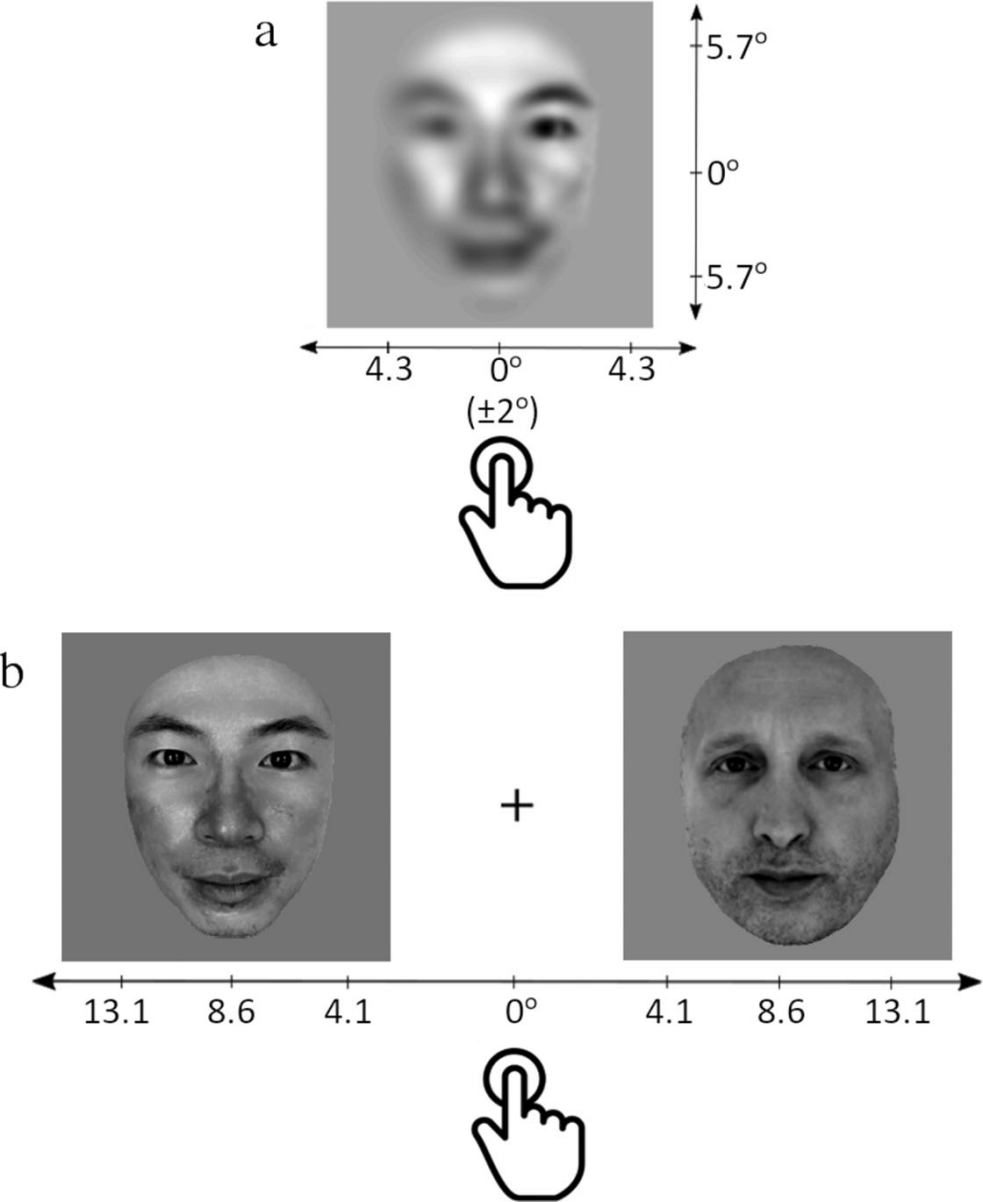
Study 2 involved the same stimuli used in study 1; however, the foveal presentation task used spatially filtered stimuli (a) to match the low-level spatial-frequencies available through extrafoveal vision. The extrafoveal presentation task required key presses (b) rather than saccadic responses as in study 1.

The stimuli and procedures used in study 2 were the same as in study 1; however, a high-pass spatial filter scaled by stimulus eccentricity (8.6° from the center of the face stimuli) was applied to the foveally presented stimuli to reconstruct the loss of visual acuity observed in extrafoveal vision (Targino Da Costa & Do, 2014). The scaling of the retinal filter replicated the spatial frequencies available for processing in proportion to the distance from the fovea, where the furthest areas of the face stimuli contained lower spatial frequencies than those closer to the central visual field (Figure 2a). Apart from the use of filtered stimuli, the procedure for the foveal key press task remained the same as in study 1. Similarly, the procedure for the extrafoveal paradigm remained unchanged from study 1, with the exception that instead of participants making eye-movements to the target race defined at the beginning of each 100 trial block, participants were instructed to press the “s” key if the target race was on the left and the “k” key if it was on the right. Thus, the laterality of the key response spatially matched that of the target in each trial. The timing of the stimulus presentation was kept the same as in study, 1 with the exception that the face images remained on the screen until a key press was made, as piloting suggested that participants required more time to categorize race than when saccadic responses were used.

Behavioral data analyses for the key press responses in both tasks were the same as for study 1 so that the behavioral results from study 1 could be statistically compared with those of study 2. Two independent samples of participants were tested in experiments 1 and 2, because these experiments were conducted in succession as the project and research aims unfolded.

To complement the mean reaction time analyses and to more thoroughly scrutinize the apparent speed advantage of saccadic responses in the extrafoveal presentation task, minimum reaction times were calculated in the saccadic response and key press response tasks to determine the earliest time that participants were able to reliably categorize the race of the faces in these respective response modalities. The minimum reaction time was calculated for each participant through χ^2^ tests using 10-ms time bins across the trials. The minimum reaction time bin was defined as the first bin where the number of correct trials statistically outperformed the number of incorrect trials, followed by three consecutively outperforming bins (*p* < .05) (Besson et al., 2017), or when a participant made no error responses in the early latency range three correct responses were required to constitute a minimum reaction time bin (Kirchner & Thorpe, 2006). The resulting minimum reaction time for each combination of the race of the target face, race of observer, and response modality were statistically compared. Finally, to explore the accuracy of the race categorization judgements in the extrafoveal saccadic choice and key press tasks, we analyzed the proportion of correct responses between conditions, including task as a factor.

## Results from study 1: Foveal and extrafoveal race categorization

### ORCA reaction time

#### Foveal key press task

**Figure 3.**
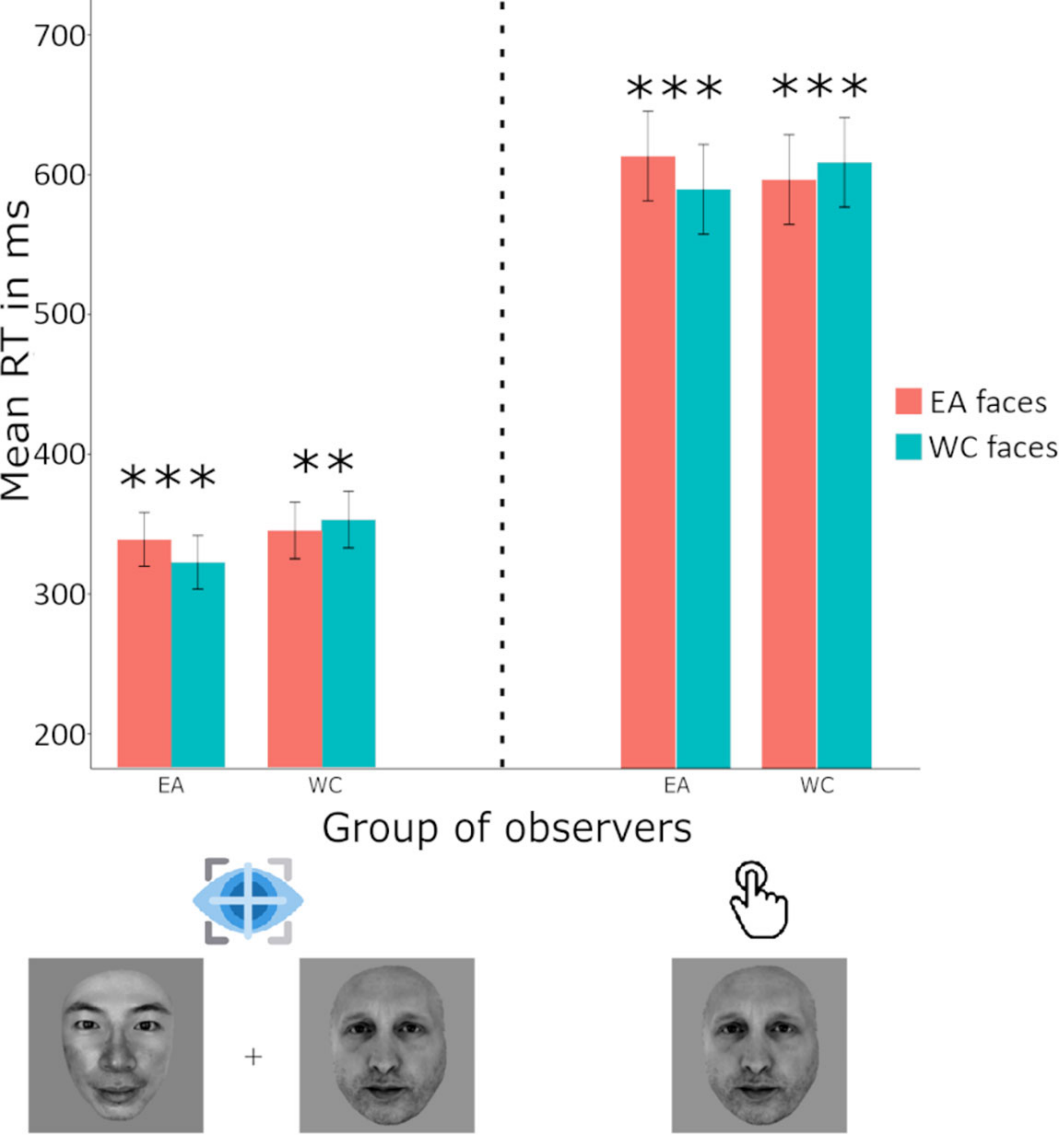
Both WC (Swiss) and EA (Japanese) observers were faster to categorize the presented faces by race when the target faces were of a different race to their own. Reaction times in the extrafoveal saccadic response task (left) were approximately 300 ms faster than when participants had to judge the race of a single face with a key press (right). ***p ≤ 0.001, **p ≤ 0.01, *p ≤ 0.05, error bars reflect 95% confidence intervals of the means.

The fitted model on key press reaction time revealed a significant main effect of the race of stimuli and a significant interaction between the group of observers and the race of stimuli (Figure 3), with β*_stimuli_asian_* = 0.03, 95% CI, 0.02–0.05, *t*(15034) *=* 4.65, *p* < 0.0001 and β*_group_asian,stimuli_asian_*= –0.10, 95% CI, −0.12 to −0.08, *t*(15034) *=* −9.5, *p* < 0.0001 . Our data showed that, on average, WC observers were faster to categorize Asian faces (M = 596 ms) compared with Caucasian faces (M = 609 ms) and EA observers were faster to categorize Caucasian faces (M = 589 ms) compared with Asian faces (M = 613 ms), whereas overall participants were 5 ms faster to categorize WC faces. No main effect was found for the group of *observers* with β_group_asian_ = 0.08, 95% CI, −0.05 to 0.21, *t*(15034) = 1.19, *p* > 0.05.

#### Extrafoveal saccadic choice task

The fitted model on reaction time (seconds) revealed a significant main effect of the race of stimuli and a significant interaction between the group of observers and the race of stimuli (Figure 3), with β*_stimuli_asian_* = 0.06, 95% CI, 0.01–0.11, *t*(9152) *=* 2.59, *p* < 0.001 and β*_group_asian,stimuli_asian_*= –0.21, 95% CI, −0.27 to −0.14, *t*(9152) *=* *–*6.20, *p* < 0.0001 . Our data showed that, on average, WC observers were faster to categorize Asian faces (M = 344 ms) compared with Caucasian faces (M = 352 ms) and EA observers were faster to categorize Caucasian faces (M = 322 ms) compared with Asian faces (M = 338 ms). The WC faces were categorized on average 4 ms faster than EA faces. No main effect was found for the group of observers with β_group_asian_ = 0.11, 95% CI −0.22 to 0.44, *t*(9152) = 0.65, *p* > 0.05.

### Fixation analysis

#### Foveal key press task

**Figure 4.**
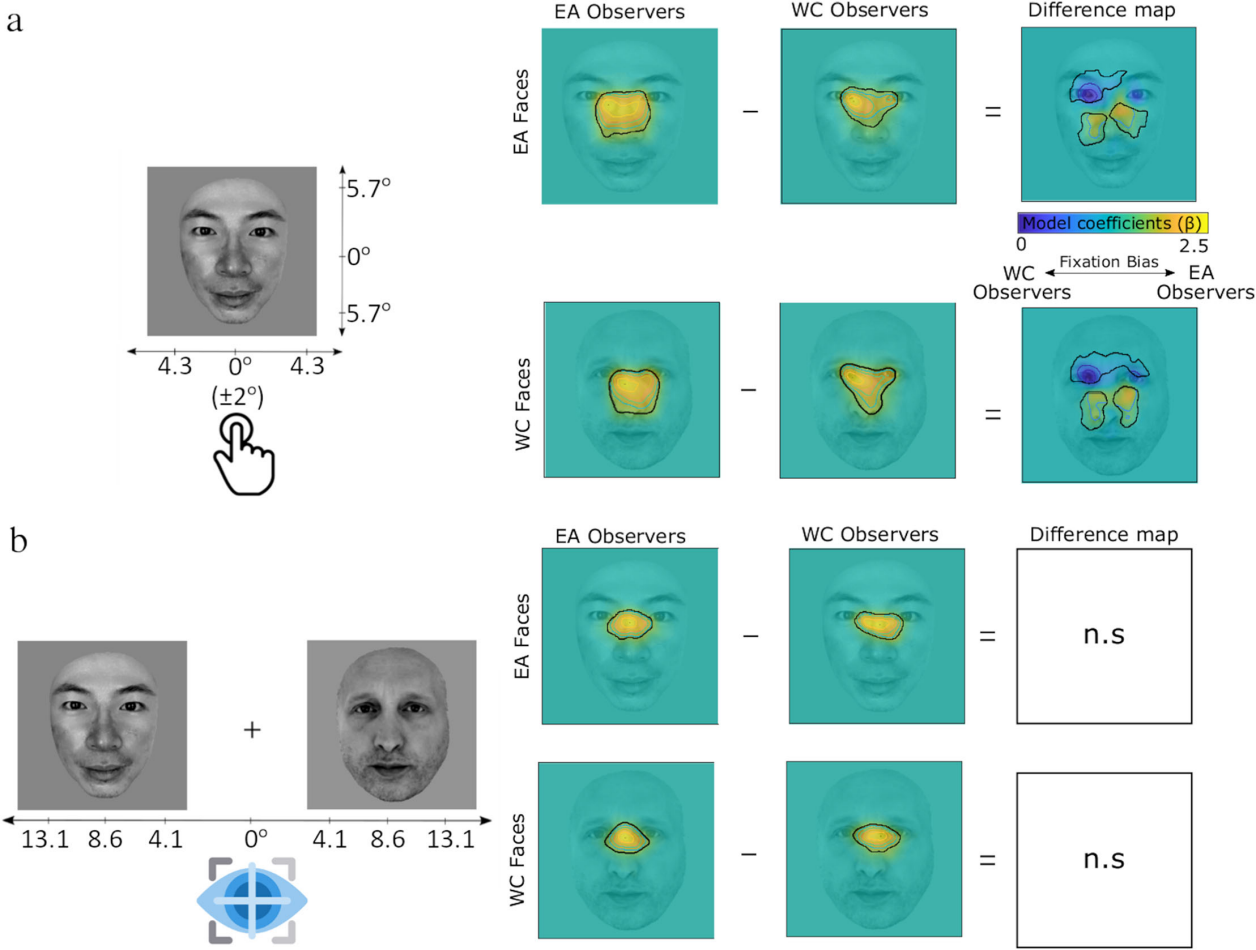
Fixation maps showing salient regions for WC (Swiss) and EA (Japanese) observers during face-categorization by race in the foveal key press task (a) and extrafoveal saccadic choice task (b). Significant areas are demarcated with black lines. Differences between the observer groups were found only in the foveal key press task, and not in the extrafoveal saccadic choice task.

Our first fitted model revealed a significant effect of the group, denoting significant differences in where the WC and EA participants tended to fixate during this task. We obtained three significant clusters for the WC faces: An eyes cluster, F(1,156) = 15.23 at the local maximum within the cluster with a beta contrast equal to −0.32, 95% CI, −0.49 to −0.16; and F(1,156) = 3.90, *p* <0.05 at the local minimum within the cluster with a beta contrast equal to −1.17 95% CI, −2.34 to 0; a left noise cluster: F(1,156) = 44.90 at the local maximum within the cluster with a beta contrast equal to 0.75, 95% CI, 0.53 to 0.97; and F(1,156) = 3.91, *p* <0.05, at the local minimum within the cluster with a beta contrast equal to 0.83, 95% CI, 0 to 1.65; and a right noise cluster: F(1,156) = 23.86 at the local maximum within the cluster with a beta contrast equal to 1.66, 95% CI, 0.99 to 2.33 and F(1,156) = 3.91, *p* < 0.05 at the local minimum within the cluster with a beta contrast equal to 0.96, 95% CI, 0 to 1.91. Similar significant clusters were found for the EA faces; an eyes cluster: F(1,156) = 18.55 at the local maximum within the cluster with a beta contrast equal to −0.73, 95% CI, −1.06 −0.39; and F(1,156) = 3.93, *p* <0.05 at the local minimum within the cluster with a beta contrast equal to −0.64 95% CI, −1.28 to 0; a left noise cluster: F(1,156) = 44 at the local maximum within the cluster with a beta contrast equal to 1.14, 95% CI, 0.80 to 1.47; and F(1,156) = 3.91, *p* <0.05, at the local minimum within the cluster with a beta contrast equal to 1.08, 95% CI, 0 to 2.16; and a right noise cluster: F(1,156) = 18.89 at the local maximum within the cluster with a beta contrast equal to 1.80, 95% CI, 0.98 to 2.61 and F(1,156) = 3.90, *p* < 0.05 at the local minimum within the cluster with a beta contrast equal to 1.09, 95% CI, 0 to 2.18. These results indicate that the WC observers' tended to sample the eye region, whereas the EA observers sampled the central region of faces, see difference maps in Figure 4a.

#### Extrafoveal saccadic choice task

In contrast with the foveal key press task, no group effects were found in the fixation analysis in the extrafoveal saccadic choice task, with no differences observed in where the WC and EA participants tended to fixate in either the WC or the EA stimuli faces (Figure 4b).

## Results from study 2: Effects of spatial filtering, face location, and response modality

### ORCA reaction time: Spatially filtered versus broadband foveal faces

**Figure 5.**
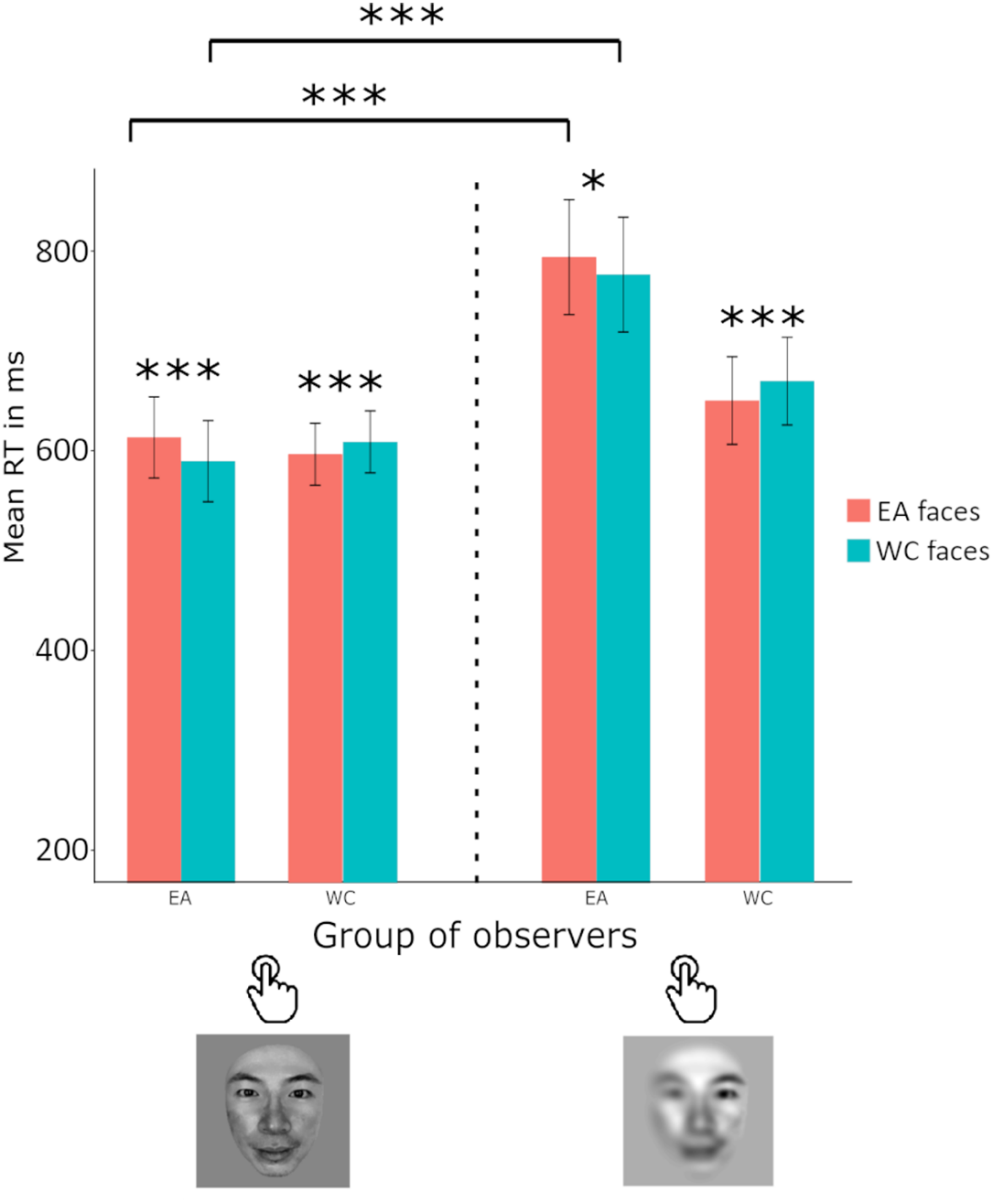
WC (Swiss) and EA (Japanese) observers were faster to categorize other-race faces than their own, however only Japanese observers exhibited an effect of stimulus quality, with slower responses to retinal-filtered faces in general. ***p ≤ 0.001, **p ≤ 0.01, *p ≤ 0.05, error bars reflect 95% confidence intervals of the means.

The fitted model on reaction time (seconds) revealed a significant main effect of the *race of* stimuli*,* the interaction between the group of observers and the race of stimuli, and the interaction between the group of observers and the task with β*_stimuli_asian_* = 0.03, 95% CI, 0.02 to 0.05, *t*(22427) *=* 4.23, *p* < 0.0001, β*_group_asian,stimuli_asian,_* = − 0.10, 95% CI, −0.12 to −0.07, *t(*22427) *=* −8.65, *p* < 0.05 and β*_group_asian,task_foveal_filtered_*= − 0.27, 95% CI, −0.51 to −0.04, *t*(22427) *=* −2.28, *p* < 0.05. As found in study 1, participants were faster to categorize the WC faces than the EA faces, although the average advantage was only 2 ms. Although both groups exhibited a typical ORCA pattern, with faster responses to other-race faces, only the EA observers exhibited an effect of stimulus quality, where the retinal-filtered stimuli led to significantly slower responses than broadband faces (Figure 5).

### ORCA reaction time: Extrafoveal versus foveal key press tasks

**Figure 6.**
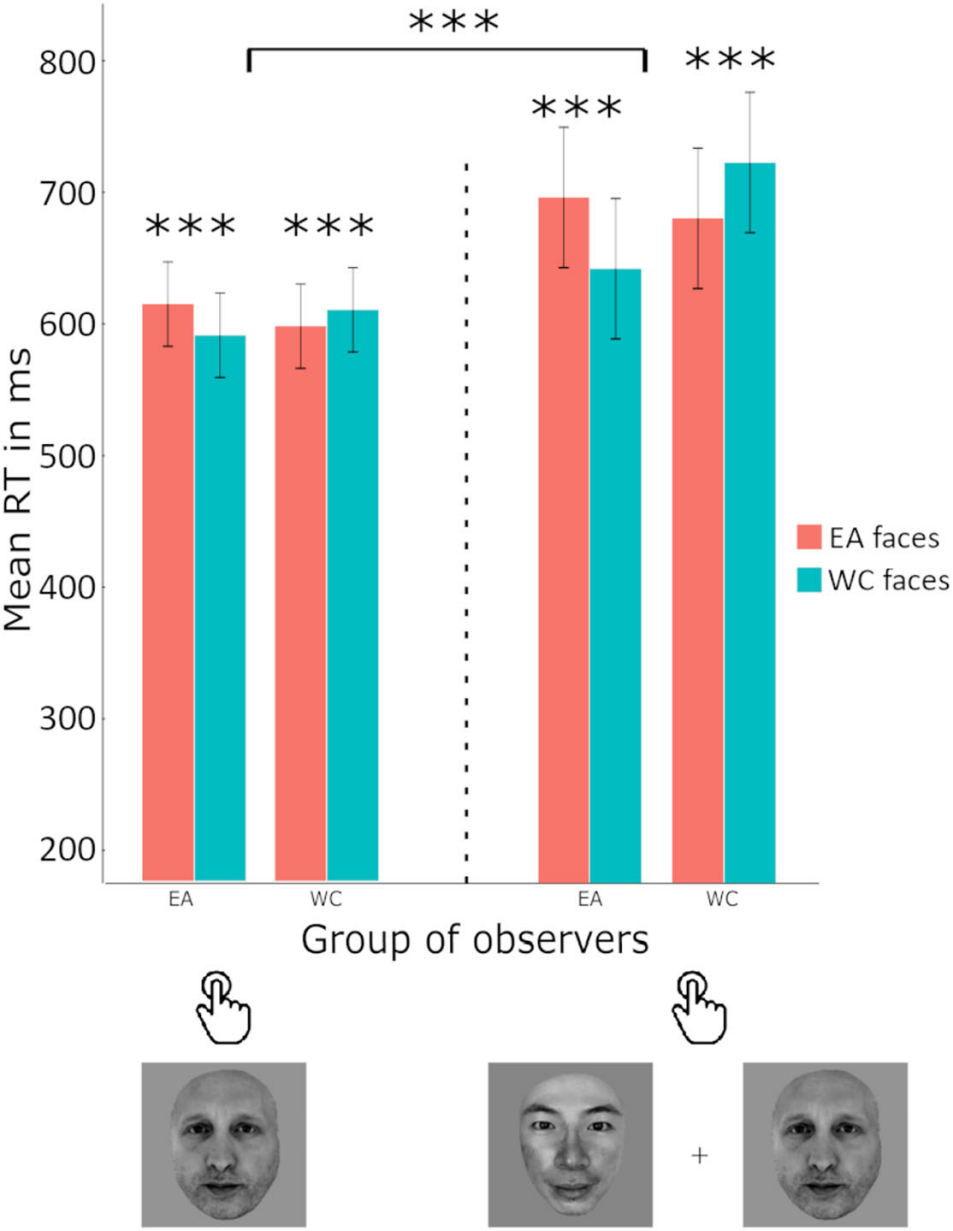
WC (Swiss) and EA (Japanese) observers were faster to categorize other-race faces than their own in both the foveal and extrafoveal key press tasks, however mean response times were significantly slower when participants categorized the race of extrafoveally presented faces. ***p ≤ 0.001, **p ≤ 0.01, *p ≤ 0.05, error bars reflect 95% confidence intervals of the means.

The fitted model on reaction time (seconds) revealed a significant main effect of the *race of stimu*li, the task, the interaction between the group of observers and the race of stimuli and the interaction between the race of stimuli, the group of observers and the task with β*_stimuli_asian_* = 0.03, 95% CI, 0.02 to 0.05, *t*(22665) *=* 4.40, *p* < 0.001, β*_task_extrafoveal_keypress_* = −0.29, 95% CI, −0.45 to −0.13, *t*(22665) *=* −3.58, *p* < 0.001, β*_group_asian,stimuli_asian_*= −0.09, 95% CI, −0.12 to −0.07, *t*(22665) *=* −9.00, *p* < 0.001 and β*_group_asian,stimuli_asian_task_extrafoveal_keypress __*= −0.10, 95% CI, −0.14 to −0.07. WC faces were categorized on average 6 ms faster than EA faces overall. An ORCA was observed in both observer groups, as were significantly faster responses to the foveally presented faces than the extrafoveally presented faces (see Figure 6).

**Figure 7.**
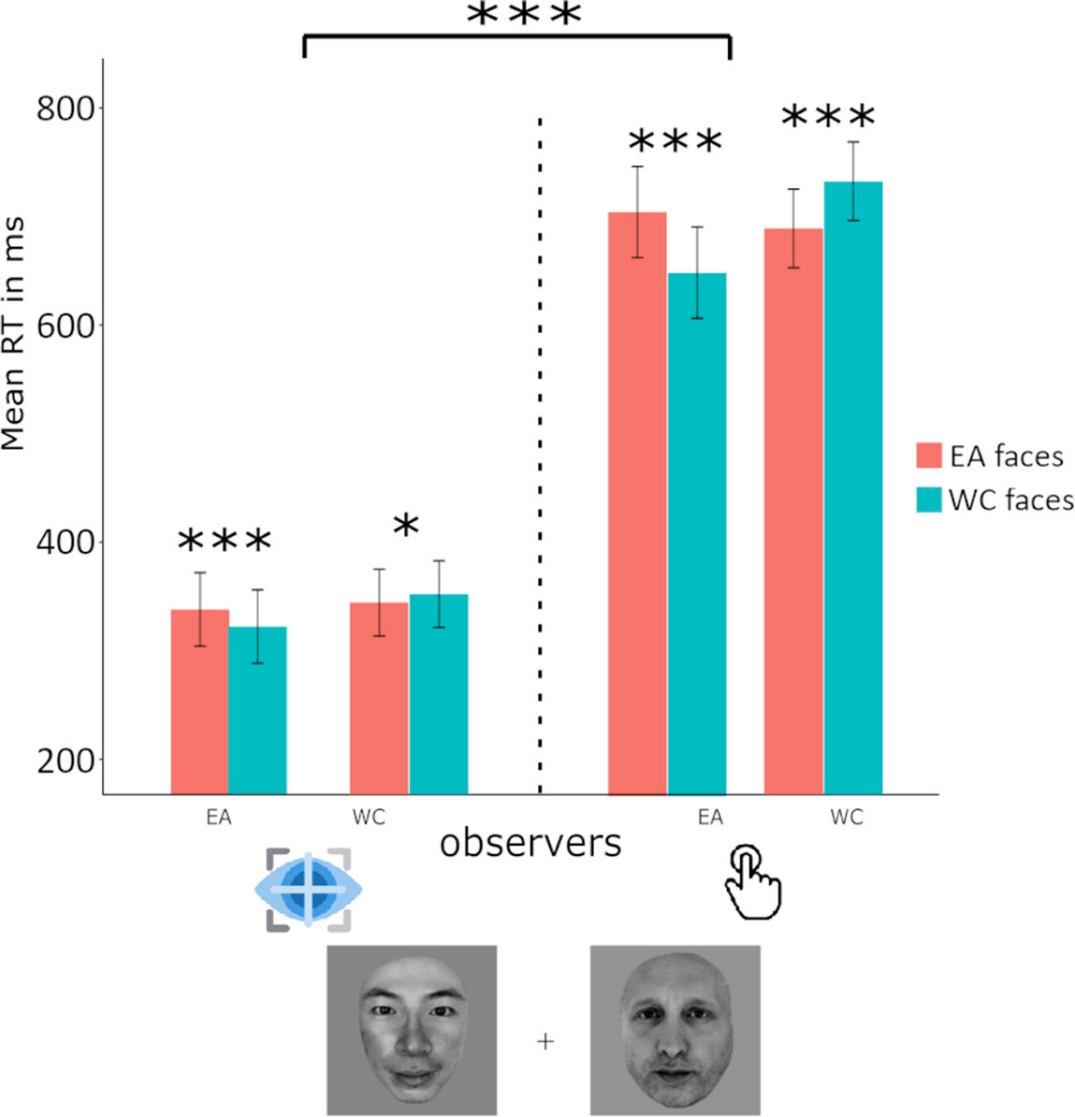
WC (Swiss) and EA (Japanese) observers were faster to categorize by race other-race than same-race faces. A comparison or response modality in the extrafoveal choice-paradigm revealed significantly faster responses when participants were instructed to saccade to the target faces than when they had to make key presses corresponding to the side of the target. ***p ≤ 0.001, **p ≤ 0.01, *p ≤ 0.05, error bars reflect 95% confidence intervals of the means.

### ORCA reaction time: Key press versus saccadic choice in extrafoveal presentation

The fitted model on reaction time (seconds) revealed a significant main effect of the race of stimuli, the task, and the interaction between the group of observers and the race of stimuli with β*_stimuli_asian_* = 0.06, 95% CI, 0.02 to 0.10, *t*(16783) *=* 2.83, *p* < 0.001, β*_task_extrafoveal_keypress,_* = −1.68, 95% CI, −2.04 to −1.33, *t(*16783) *=* −9.26, *p* < 0.001 and β*_group_asian,stimuli_asian_*= −0.21, 95% CI, −0.27 to −0.15, *t*(16783) *=* −6.80, *p* < 0.001. WC faces were categorized on average 5 ms faster than EA faces overall. An ORCA was observed in both observer groups, as were significantly faster responses in the saccadic choice modality than in the key press modality (see Figure 7).

### Minimum reaction time: Key press versus saccadic choice in extrafoveal presentation

**Figure 8.**
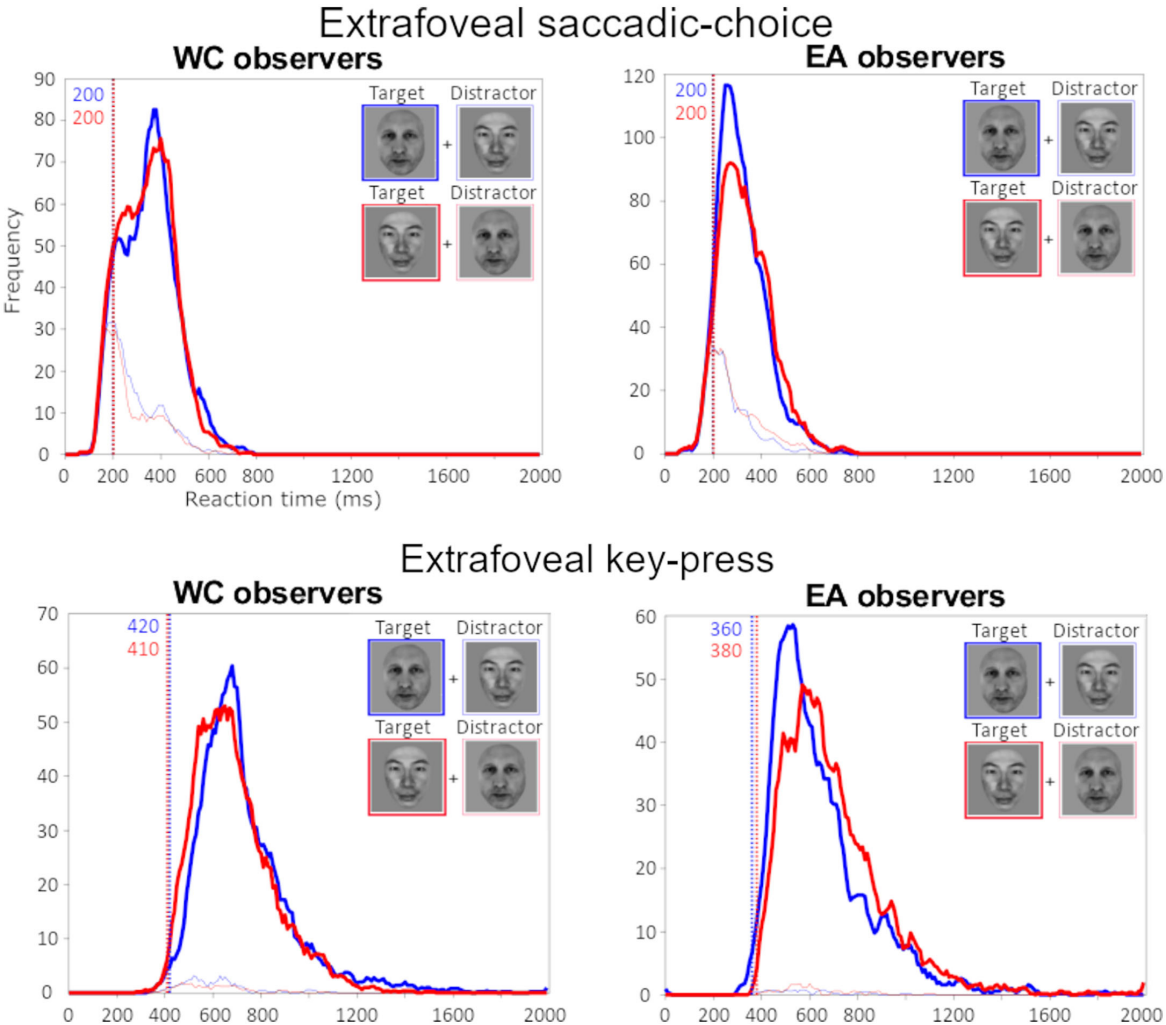
WC (Swiss) and EA (Japanese) observers were able to reliably categorize the faces by race in the extrafoveal saccadic choice task within 200 ms of stimulus presentation, suggesting rapid processing and response preparation in race perception.

**Figure 9.**
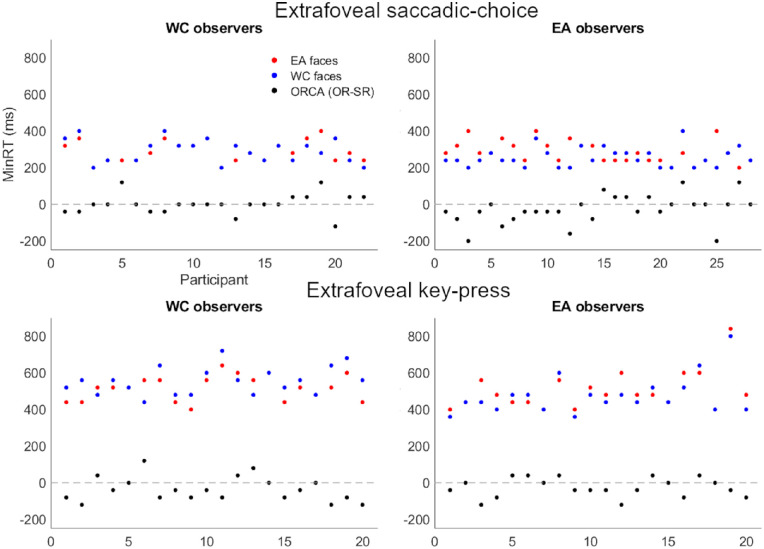
The minimum reaction time analyses of the individual participants suggested a consistent speed advantage for saccadic choice responses compared with key press responses. ORCA value below.

An analysis of the minimum saccadic reaction times revealed that race was able to be reliably categorized within 200 ms after stimulus presentation for both WC and EA observers (Figure 8). In contrast, a minimum reaction time analysis of the key press responses to the same extrafoveal presentation revealed a much slower minimum response time, taking almost twice as much time to make reliable race categorization judgements (392 ms). An additional minimum reaction time analysis on individual participant data showed a similar overall pattern of faster responses in the saccade choice modality than the key press modality (Figure 9).

### ORCA accuracy: Key press versus saccadic choice in extrafoveal presentation

**Figure 10.**
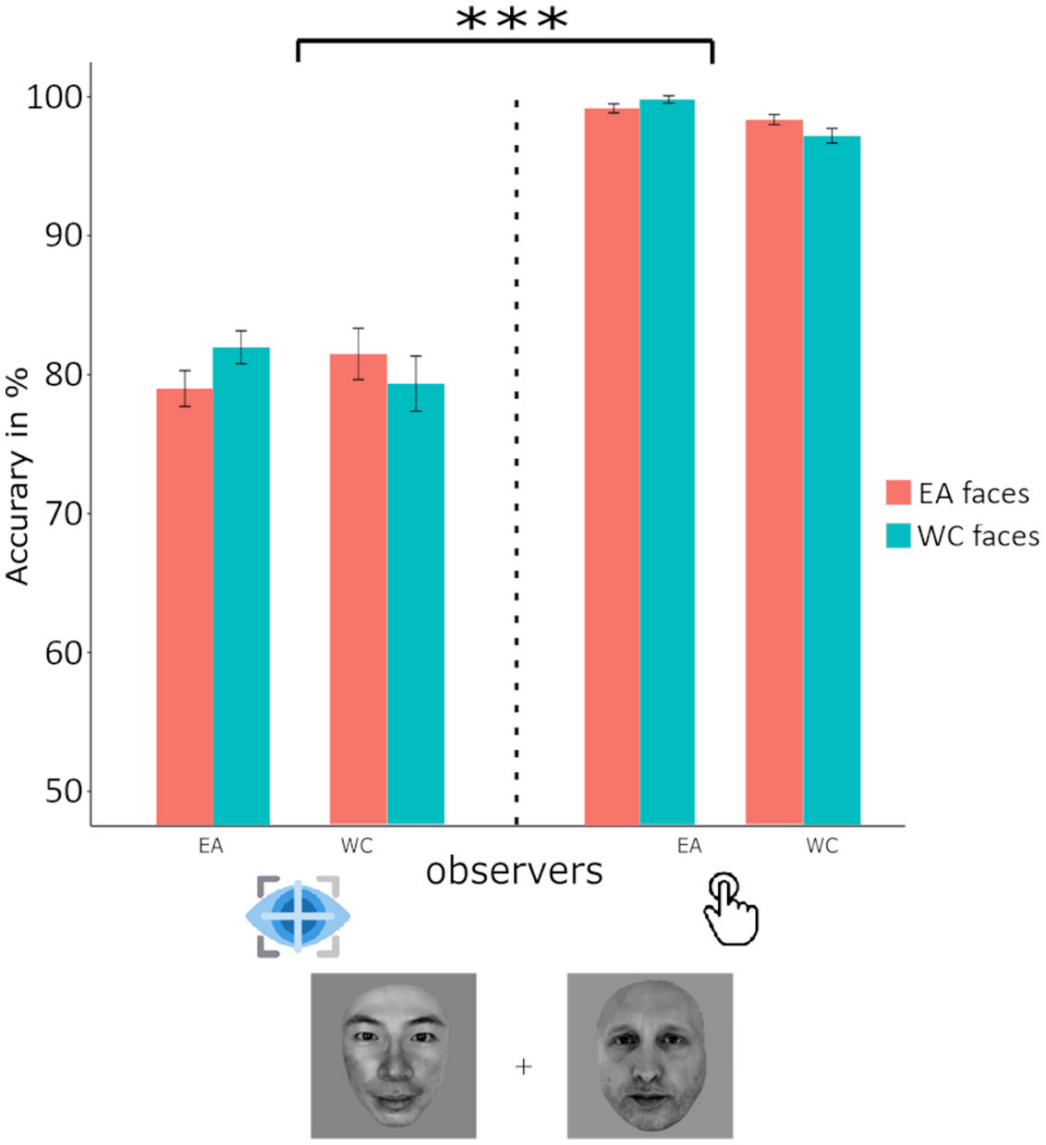
WC (Swiss) and EA (Japanese) observers did not differ for the accuracy in categorizing by race other- than same-race faces. A comparison or response modality in the extrafoveal choice paradigm revealed significantly lower accuracy when participants were instructed to saccade to the target faces than when they had to make key presses corresponding with the side of the target. ***p ≤ 0.001, **p ≤ 0.01, *p ≤ 0.05, error bars reflect 95% confidence intervals of the means.

The fitted mixed model with binomial family on the correct response revealed a significant main effect of the race of stimuli, task, the interaction between the group of observers and the race of stimuli and the interaction between the group of observers and the task, with β*_stimuli_asian_* = 0.13, 95% CI, 0.002 to 0.27, *z =* 2, *p* < 0.05, β*_task_SRTkeypress_* = 2.12, 95% CI, 1.74 to 2.50, *z = 10.95*, *p* < 0.0001, β*_task_SRTkeypress,stimuli_asian_*= −0.31, 95% CI, −0.50 to −0.14, z *=* −3.42, *p* < 0.0001 and β*_task_SRTkeypress,group_asian_*= 0.82, 95% CI, 0.21 to 1.44, z *=* 2.64, *p* < 0.001. The critical comparison of task revealed a clear difference in accuracy between the saccadic choice and key press response modalities, with an average accuracy of 80% in the former and 98% in the latter (Figure 10). Overall participants were more accurate at categorizing the EA stimuli.

## Discussion

The results of study 1 highlight the robustness of the ORCA in foveated key pressed tasks. However, the novelty lies in the majority of observers reliably categorizing faces by race when presented extrafoveally in the saccadic choice task. A full crossover interaction was observed between WC (Swiss) and EA (Japanese) observers, reinforcing the universal (cross-cultural) nature of the ORCA. Cross-cultural differences in the fixation analysis were evident only in the foveal key press task, with WC observers spending more time fixating the eye regions of the faces, whereas EA spent more time fixating the central nose region, regardless of the race of the face stimuli. Such a pattern echoes previous findings during a race categorization task (Blais et al., 2008). When interpreting the absence of a similar effect in the saccadic choice paradigm, it is relevant to consider that in this task the race categorization decision was made before a fixation being made on the face. However, the landing positions observed in our study correspond closely with those found by Or et al. (2015), centering on the bridge of the nose and interpreted in that study to reflect an optimal point for efficiently sampling faces when constrained to a limited number of fixations. Alternatively, the cross-cultural intermediate fixation location might represent a center-of-gravity effect (Bindemann et al., 2009), where the geometric upper-middle portion of the faces served as the optimal landing location for participants to express their decision in the task, where a further visual analysis was no longer necessary. However, because it is quite likely that participants in our study used the initial fixation on the faces to confirm that they made a saccade to the correct race face, we favor the interpretation that the upper-central landing points of fixations found in our study reflect an optimal location for visual sampling akin to that found by Or et al. (2015).

Aside from the speed advantage for other-race faces observed in the extrafoveal saccadic choice task, participants were able to achieve this task surprisingly faster than expected. Although a direct comparison of the foveal key press and extrafoveal saccadic response tasks is problematic owing to a difference in both the response modality and the low-level visual differences between the tasks, participants were nevertheless approximately 260 ms faster to categorize race in the extrafoveal saccadic response task (approximately 340 ms) than in the foveal key press task (approximately 600 ms). These results provide a clear indication that race is processed very quickly after face presentation, even outside of the fovea. This observation raised the question of whether the overall speed advantage we observed for categorizing race extrafoveally was related to the impoverished visual quality of the face stimuli owing to the loss of acuity inherent in extrafoveal vision. The follow-up experiment in study 2 directly addressed this question by comparing the speed of race categorization for foveally presented faces that had been retinally filtered to approximate extrafoveal acuity with broadband faces. Although a robust ORCA effect was evident in both paradigms, this second study showed that the saccadic responses were on average twice as fast as key press responses (approximately 340 ms and approximately 680 ms, respectively) (Figure 10). A similar pattern was observed in the minimum reaction time analysis, where reliable race categorization responses were evident within 200 ms of stimulus presentation in the saccadic choice paradigm compared with 392 ms in the key press paradigm (Figure 8). This effect was not exaggerated compared with the full-spectrum conditions, as observed in a previous study (Zhang et al., 2017). This discrepancy might be accounted for by the more ecologically valid retinal filter used in our study (Targino Da Costa & Do, 2014), controlling for visual eccentricity. Overall, it is clear that race categorization saccadic responses were significantly faster than key press responses, even when the visual presentation paradigms were matched in terms of both visual quality and competition.

## General discussion

The purpose of this series of studies and experiments was to determine whether observers are able to extract race in extrafoveal vision, and whether other-race faces produce faster categorization responses than same-race faces, as previously found in ORCA studies using foveal presentations. We observed consistent, full-crossover interaction effects for the ORCA in all our experiments, confirming the robustness of this perceptual phenomenon. Our data clearly show fast and effective extrafoveal face categorization by race. Strikingly, the speed at which extrafoveal race categorization occurred in the current study was much faster than anticipated. Reliable race categorization occurred within 200 ms of face presentation outside of the fovea and this speed advantage cannot by accounted for by differences in spatial frequency properties per se.

Such timing is in line with electrophysiological studies showing early race categorization effects with foveated faces (Caharel et al., 2011; Ito & Urland, 2003; Stahl et al., 2010; Vizioli et al., 2010; Walker et al., 2008; Wiese, 2013; but see Caldara et al., 2003; Caldara et al., 2004; Lv et al., 2015; Sun et al., 2014; Tanaka & Pierce, 2009; Wiese et al., 2009), confirming the solidity of the current eye movement method as a sensitive and effective tool to investigate the temporal dynamics of categorical events.

As noted in the preceding section, the comparison of saccadic choice responses with key presses when categorizing race in extrafoveal faces showed a much faster categorization when eye movements were used as the response medium. Coupled with the finding that response times for extrafoveal key presses were actually slower than key presses to foveally presented faces, it is clear that the speed advantage was related to the response modality and not specifically an extrafoveal perception of the faces. Such a finding is relevant in a number of ways. Primarily as a methodological concern, the reaction time can give an insight into the end of a cascade of perceptual and decisional processes and establish a time frame for the latest stages at which these processes occur. In line with our findings, previous studies have found race to be categorized reliably within approximately 600 ms when using conventional key presses as a response modality (Valentine & Endo, 1992, mean reaction time = 576 ms; Caldara et al., 2004, mean reaction time = 555 ms, Zhao & Bentin, 2008, 2011, mean reaction time = 597 ms and 662 ms, respectively). When inferring the processes relating to the categorization of race, such a time range may involve both early and later stages of visual processing to perform such a task. However, in light of the current saccadic choice reaction time results that race can be reliably categorized extrafoveally within 200 ms, it is clear that it is very likely that the early perceptual processing stages are recruited for the processing of race. To relate this to a general methodological point, these results thus suggest that the use of saccadic responses as a response modality may provide a clearer functional signature of how fast other types of processing may occur as well, outside of the realm of face–race processing.

Interestingly, the expected cultural bias (Blais et al., 2008) observed in the visual sampling strategies during face recognition and categorization by race appeared only when faces were foveated. In this condition only, Westerners fixated more to the eye region compared with the Easterners, who fixated more the central nose region. This observation offers an interpretation of the apparent absence of cultural fixation biases with very constrained experimental designs (Or et al., 2015). WCs and EA observers shared a similar fixation landing location between the bridge of the eyes only in the constrained extrafoveal conditions, in which information intake needs to be maximized after the first fixation. However, the processing of faces in extrafoveal vison and the inability to cancel a saccadic movement beyond a critical time would predict larger error rates. In fact, the comparison of error rates between the saccadic choice and key press tasks showed that accuracy in the key press task approached ceiling with an average of 98%, this decreased significantly in the saccadic choice task to 80%. Altogether, these data show that the fixation strategy used with foveated faces is slower, culturally dependent, but optimal for face recognition and categorization by race.

Although cultural differences in race categorization was not the main focus of the current series of experiments per se, it is of note that the spatial frequency manipulation used in study 2 seemed to impact EA observers much more than WC observers. Previous studies investigating cultural differences in face processing tasks have typically found that EA participants tend to rely more heavily on low spatial frequencies, whereas WC participants make more relative use of high spatial frequencies (Caldara, Zhou, & Miellet, 2010; Estéphan et al., 2018; Miellet et al., 2010; Tardif et al., 2017). According to these findings, we might have expected a decrease in high spatial frequencies to disproportionately delay responses in the WC participant sample. The opposite pattern was observed, however, with significantly slower response times in the EA participant sample when the face stimuli had been filtered spatially to decrease high frequencies (Figure 5). These previous findings related to face recognition tasks, so it is therefore not clear that such a cultural-dependent pattern would directly apply to race categorization. However, given the previous finding that race categorization relies heavily on the perception of low spatial frequencies (Zhang et al., 2017), our current findings are somewhat unexpected, and, if they are robust, this dimension would require further consideration in future studies. Separate from cultural differences in visual sampling during race categorization, we observed a consistent advantage in reaction time for WC faces compared with EA faces, regardless of the task or observer group. However, as this effect was very small, ranging from 2 ms to 6 ms, and is not readily explained theoretically or by low-level visual differences, we will refrain from speculating on the source of the effect.

As suggested by theoretical (Valentine, 1991; Valentine & Endo, 1992) and computational face–space models (Balas, 2013; Caldara & Abdi, 2006; Furl et al., 2002; O'Toole et al., 1994), with visual expertise, the decreased frequency of encountering distinctive features of a particular race may lead to an increase for their saliency. This experience-based process might lead to a greater sensitivity of the visual system to detect them. The speed at which race was detected (approximately 200 ms) might be a functional signature and a byproduct of a tuning toward same-race faces. As such, the sensitivity to race in extrafoveal vision is likely to be specifically modulated by experience with different races and future studies are necessary to clarify this issue, as well as how cultural differences in spatial frequency use during race categorization develop and interact in paradigms that intrinsically involve modulation of these low-level factors such as in extrafoveal perception. Overall, the greater sensitivity to the visual features shared by other-race faces may thus lead to early perceptual differences in face processing, interacting with face detection and later decisional processes. Such an effect would have strong implications in contexts where faces in crowds are scanned by security or police enforcement agencies. The bias found for people to misidentify objects as weapons more frequently when primed by other-race faces (Payne, 2001) relies on the quick extraction of race from prime faces preceding object images. The current results suggest race to be extracted in the very early stages of perceptual processing, and so effects observed in the aforementioned study may occur as part of a cascade of processes where object recognition (or indeed misperception) is influenced by the initial face processing stages. Such an effect may have strong implications for interpreting asymmetries in police use of deadly force applied to people of minority races (Edwards, Lee, & Esposito, 2019). Simulations of threatening and nonthreatening contexts involving firearms has revealed a tendency for people to fire more frequently on unarmed people of another race in random participant samples (Correll et al., 2002) as well as samples of police officers (Plant & Peruche, 2005). The latter study found that training eliminated this effect, which suggests that such race-related effects are not bound to the perception of other-race faces themselves, but are more likely to be due to preexisting attitudes or beliefs related to such groups. Such a result points to an important conceptual and practical separation between the early perceptual face processing relating to expertise and familiarity, and the potential consequences of these processes as they interact with existing beliefs or biases. The question of how early perceptual processing of race may interact with explicit or implicit task demands raises further questions in light of the results of the current study, such as whether race is sufficiently salient in extrafoveal vision that it may influence other types of recognition or categorization even when it is unrelated to the goals? What are the consequences of such early categorization of race in terms of other types of analysis that may interact with existing beliefs and attitudes? Such questions are raised by the current results and require future attention, as there is still a lot we do not yet know about our sensitivity to the unfamiliar when it comes to race.

## Conclusions

Saccades are the fastest movement our body can perform. Our data show that the speed of race categorization is boosted by eye movements toward visual field eccentricity. This early visual categorization process eliminates fine-grained and time-consuming information processing and might represent the entry level of a cascade of social evaluation judgements. Saccade responses were not as accurate compared with foveated button responses, but nevertheless reached a significantly high level of efficiency. Crucially, altogether our observations provide new evidence of race as a powerful rapid low-level visual signal transmitted by faces, which could be decoded outside central vision. This rapid visual categorization could relate to primitive functional mechanisms dedicated to the evolutionary-relevant social categorization of ingroup/outgroup members.
